# Perceptual representations in L1 and L2 spatial and abstract language processing: applying an innovative sentence-diagram verification paradigm

**DOI:** 10.3389/fnhum.2024.1425576

**Published:** 2024-10-15

**Authors:** Menghan Wang, Helen Zhao

**Affiliations:** School of Languages and Linguistics, Faculty of Arts, The University of Melbourne, Melbourne, VIC, Australia

**Keywords:** perceptual representations, bilingual processing, mental imagery, schematic diagrams, semantic abstractness

## Abstract

**Introduction:**

Perceptual representations in language comprehension were examined using sentence-picture verification tasks. However, concerns have been raised regarding the suitability of concrete pictures for representing abstract concepts compared to image-schematic diagrams. To assess the perceptual representations of spatial and abstract domains in both first language (L1) and second language (L2) processing, the study tests bilingual speakers’ mental imagery on the basis of the simulation-based L1 comprehension model and proposes a simulation-based L2 comprehension model, supported by empirical evidence from an innovative sentence-diagram verification paradigm.

**Methods:**

41 adult L1 Mandarin Chinese speakers participated in the study. 21 participants completed the Chinese sentence-diagram verification task (Experiment 1), while 20 participants completed the translation-equivalent version in L2 English (Experiment 2). Participants read a sentence [e.g., *A diligent worker walked into the office (spatial sense); A strong team headed into the final (abstract sense)]* at their self-paced speed, followed by a congruent (e.g., *into diagram*) or incongruent diagram (e.g., *out-of diagram*), and made binary judgments to verify spatial configurations between the sentence and diagram. Semantic rating tasks in both Chinese and English were also conducted to validate congruency between diagrams and sentences in both languages.

**Results and discussion:**

Results from Experiment 1 indicate overall compatibility effects on L1 Chinese processing, unaffected by directional verbs or abstractness of sense. Results from Experiment 2 reveal interference effects on L2 English processing, with interference observed only after reading sentences encoding spatial senses, not abstract senses. Aligning with previous findings using sentence-picture verification tasks, the current findings confirm the weaker mental simulation effects in L2 processing compared to L1 processing. These findings extend the existing simulation-based L1 comprehension model, provide empirical support for the proposed simulation-based L2 comprehension model, and validate the innovative sentence-diagram verification paradigm for examining image-schematic representations in spatial and abstract language processing among Chinese-English bilinguals. The paradigm holds significant potential for research on perceptual representations in processing a broader range of grammatical and semantic properties during both online and offline L1 and L2 comprehension.

## 1 Introduction

Embodied cognition, a fundamental theory in cognitive linguistics, posits that human cognition and language are grounded in perceptual experiences and shaped through bodily interactions with the world (Johnson, 1987; Lakoff, 1987; Barsalou, 1999, 2008; Evans and Green, 2006). It challenges the traditional view that language processing involves the manipulation of abstract symbols, proposing instead that it relies on the activation of mental imagery associated with the meaning of sentences or utterances (Zwaan, 2014). The cognitive process of mentally simulating actions, sensations, or spatial configurations described in a text is thought to be an integral part of language comprehension, as it connects linguistic representations to our rich perceptual and experiential knowledge (Zwaan, 2004; Bergen and Chang, 2013; Bergen, 2015).

The early embodied mental simulation theories, such as the perceptual symbol system (Barsalou, 1999, 2008) and the immersed experiencer framework (Zwaan, 2004), propose that cognition is inherently perceptual. According to these theories, our understanding of concepts emerges from integrating modal representations based on multimodal sensory-motor experiences, including vision, audition, movement, and mental states. These experiences are stored symbolically as image schemas in long-term memory, with forms that act as multimodal analogs to the referents. When encountering real-world referents, top-down memory retrieval routinely reactivates these image schemas. These theories emphasize the engagement of language comprehenders in depicted situations where linguistic input triggers their perceptual and motor representations and highlight the dynamic nature of mental representations and experiential states in language processing. While these theories strongly advocate for embodied mental simulation, they have faced criticism. They are more successful in explaining spatial language comprehension than abstract language (Wiemer-Hastings and Graesser, 1999; Zwaan, 2004; Barsalou, 2020), as abstract language often lacks perceptual and experiential grounding without concrete referents in the world (Moseley et al., 2012). Additionally, these theories prioritize the detailed mechanism of sensorimotor activation over language processing, leading to criticism for neglecting the role of linguistic input constructions in mental imagery (Bergen et al., 2007). Nevertheless, they laid the theoretical groundwork for subsequent developments in mental simulation models.

Building upon earlier theoretical frameworks of mental imagery, Bergen and Chang (2005) proposed a computational simulation model and further refined it in 2013. This model represents one of the latest simulation-based language understanding models, which divides language comprehension into three core processes (i.e., *constructional analysis*, *contextual resolution*, and *embodied simulation*). These processes are argued to overlap temporally and mutually influence each other, highlighting the dynamic nature of mental imagery. The *constructional analysis* process involves identifying the constructional information (form and meaning) instantiated by a given utterance and assembling a corresponding *semantic specification* that depicts the evoked meaning schemas and their interconnections (Bergen and Chang, 2013). The *contextual resolution* process maps objects and events in the *semantic specification* to the current communicative context, resulting in a *resolved semantic specification*. This stage activates world knowledge about entities and events in the communicative context. The third process, *embodied simulation*, involves dynamic embodied structures in the *resolved semantic specification* generating contextually appropriate inferences. According to this computational simulation model, language comprehension not only mirrors traditional syntactic parsing processes that automatically analyze the syntactic structure of a given utterance but also extends its scope to consider the specific communicative context that best situates the meaning of the utterance.

The theoretical models of mental imagery in language comprehension have established robust foundations, prompting empirical studies to validate and refine these frameworks. Most research has focused on visual and motor simulation in processing words and sentences in the first language (L1) (Bergen et al., 2003, 2007, 2010; Bergen, 2005; Bergen and Wheeler, 2005, 2010; Sato and Bergen, 2013; Liu and Bergen, 2016). However, there has been a fast-growing interest in embodied cognition in the context of second language (L2) processing over the last decade. Empirical questions have centered on understanding the accessibility of sensorimotor activation mechanisms in L2 processing and the L2-related factors that influence the interaction between sensorimotor simulation and linguistic processing. Despite the growing body of empirical evidence on L2 mental imagery, there remains a lack of an underpinning theoretical framework. Therefore, the current study aims to make an initial attempt to propose an L2 model of mental simulation, drawing on Bergen and Chang’s (2013) simulation-based L1 processing model. The findings from the current empirical study will also contribute to refining this proposed L2 model.

### 1.1 Mental imagery in first language processing

Previous studies on embodied mental imagery have explored the interaction between image schema and linguistic representations, uncovering compatibility and interference effects in the language comprehension process. The compatibility effect suggests that language processing activates perceptual neurons associated with mental representations, resulting in faster responses to corresponding images compared to incompatible ones (Stanfield and Zwaan, 2001; Zwaan et al., 2002; Bergen, 2007). For instance, when processing a sentence like *A boy climbs a mountain*, the UP-DOWN schema might be activated, leading to quicker responses to a vertical spatial configuration than to a horizontal one. In contrast, the interference effect indicates that language processing occupies the same perceptual neurons of mental representation, potentially hindering responses to corresponding images and causing delays compared to incompatible images. This phenomenon has been observed in studies where language processing interferes with the mental imagery of corresponding visual representations (Bergen, 2005; Kaschak et al., 2005; Bergen et al., 2007; Connell, 2007). These early findings establish the foundation for understanding how language comprehension involves mental imagery and how the embodied nature of cognition shapes the interpretation of linguistic expressions.

The sentence-picture verification task (SPVT) paradigm is widely used to examine mental imagery effects, often employing response time (RT) analysis (Stanfield and Zwaan, 2001; Bergen et al., 2003; de Koning et al., 2017a). In the SPVT, participants are presented with a sentence followed by a picture, and they must quickly determine whether the picture matches or mismatches the content of the preceding sentence. For instance, a seminal study by Stanfield and Zwaan (2001) utilized the SPVT to investigate compatibility effects in mental simulation related to spatial orientation. Participants read a sentence implying the orientation of a concrete object, e.g., “*John put the pencil in the drawer*” (horizontal) or “*John put the pencil in the cup*” (vertical), and viewed a picture of the object presented in either horizontal or vertical orientation. The results indicated that verification RTs were 44 milliseconds shorter in the matching condition than the mismatching condition, suggesting a compatibility effect. This implies that recognition of objects by English native speakers (NSs) was influenced by the orientation implied in the sentences. In summary, the SPVT paradigm has been pivotal in revealing the role of mental imagery in L1 processing, particularly highlighting the interplay between activated image schemas and semantic specifications.

So far, mental imagery effects have primarily been investigated in the context of L1 processing by adult NSs. Variations in these effects across studies are attributed to factors such as target languages (Sato et al., 2013; de Koning et al., 2017b; Chen et al., 2020; Bai et al., 2022), abstractness of meaning (Richardson et al., 2003; Bergen et al., 2007; Richardson and Matlock, 2007; Guan et al., 2013; Liu and Bergen, 2016), and processing capacity (Madden and Zwaan, 2006). Regarding crosslinguistic variations, Chen et al. (2020) examined whether mental simulation was affected by object size and orientation through an SPVT among L1 English, Mandarin Chinese, and Dutch speakers. Despite the similar compatibility effects of orientation identified in Chinese and English, the slower RTs and lower accuracy rates (ARs) in L1 Chinese participants underscore potential concerns about the validity of task stimuli in Chinese. Moreover, they found the effect magnitude for orientation was smaller than object size, which raises the question of whether the smaller effect was attributed to the lack of control of semantic dynamicity in orientation, given some sentences expressed a static scene (e.g., *The pen is on the table*), while some expressed dynamic movement (e.g., *The missile was flying over the sea*). This lack of consideration of dynamicity and between different L1 groups appeal for an examination of mental imagery in processing sentences that express dynamic spatial orientation (i.e., directionality) in particular and further comparisons between languages like Mandarin Chinese and English to deepen our understanding of language-specific influences.

Previous empirical findings have confirmed the controversy surrounding the applicability of embodied mental simulation theories (Barsalou, 1999, 2008; Zwaan, 2004) in abstract language processing. The existing findings are mixed, with some studies showing a comparable simulation effect in both concrete and abstract language (Glenberg and Kaschak, 2002; Richardson et al., 2003; Richardson and Matlock, 2007; Guan et al., 2013; Wang and Zhao, 2024), while others observed simulation effects only in concrete language but not in abstract language processing (Bergen et al., 2007; Bergen and Wheeler, 2010; Liu and Bergen, 2016). These mixed findings could be attributed to the different varieties of sensorimotor features being investigated in these studies, such as motion (Glenberg and Kaschak, 2002; Richardson and Matlock, 2007; Bergen and Wheeler, 2010; Guan et al., 2013; Liu and Bergen, 2016), and spatial orientation (Richardson et al., 2003) in the vertical axis (up vs. down)(Bergen et al., 2007). Because these sensorimotor features may engage different cognitive mechanisms depending on the concreteness or abstractness of the language, the inconsistencies in previous research may arise from variations in how these features interact with different types of linguistic content. Therefore, the present study focuses on mental imagery in the processing of literal and abstract language expressing spatial directionality.

### 1.2 Mental imagery in second language processing

There is a recent surge in interest in understanding how embodied mental simulation operates in L2 processing (Monaco et al., 2019; Norman and Peleg, 2022; Wang and Zhao, 2023, 2024; Chen et al., 2024; Vanek et al., 2024). Findings from L2 mental imagery studies have revealed both similarities and differences compared to L1 mental imagery patterns. Similar to observations in L1 mental imagery studies, compatibility (Tomczak and Ewert, 2015; Ahn and Jiang, 2018; Koster et al., 2018), and interference effects (Wheeler and Stojanovic, 2006; Vukovic and Williams, 2014) have been reported in the L2 context. However, certain studies also identified partial simulation (Atkinson, 2010; Foroni, 2015; Norman and Peleg, 2022) or no mental imagery effect in L2 processing (Wu, 2016; Chen et al., 2019).

Existing studies argued that L2 mental imagery is modulated by several key factors, including variations across languages and perceptual features (Koster et al., 2018; Zhang and Vanek, 2021). For example, Koster et al. (2018) investigated Spanish learners of L2 German and German learners of L2 Spanish using SPVTs. They examined orientation and size, drawing on crosslinguistic differences between German and Spanish. Their results revealed no mental imagery effects for orientation in both NSs and L2 learners. Interestingly, Spanish NSs exhibited size-related compatibility effects, while L2 Spanish learners did not, mirroring patterns observed in Dutch child speakers (de Koning et al., 2017b). These findings suggest a potential extension of L1 mental imagery effects related to size into the realm of L2, with language-specific factors modulating L2 mental imagery effects, as evidenced by the absence of a size effect in German.

L2 mental imagery can also be modulated by the abstractness of meaning. L2 mental imagery in abstract language processing might not be as intuitive and automatic as in L1. Abstract meaning could be relatively more difficult for L2 learners to comprehend compared to literal meanings (Littlemore and Low, 2006; Littlemore et al., 2011; Shi et al., 2023). Nevertheless, the investigation of L2 mental imagery in abstract language processing are very few and still controversial (Feng and Zhou, 2021; Wang and Zhao, 2023, 2024). Feng and Zhou (2021) adopted a picture priming paradigm to examine the embodiment of verbs in predicate metaphor processing in L1 Mandarin and L2 English. In the priming task, participants were presented with a related or unrelated picture prime and then read L2 English and L1 Mandarin sentences containing conventional or novel metaphors. Results showed stronger compatibility effects on processing novel predicate metaphors (e.g., *The tax pinched the industry.*) in both high-proficiency and low-proficiency L2 learner groups but weaker compatibility effects on processing conventional predicate metaphors (e.g., *The newspaper bent the truth.*) in the lower L2 proficiency group. The finding suggests the graded compatibility effects could be affected by metaphor novelty and L2 proficiency.

Wang and Zhao, (2023, 2024) adopted a semantic priming paradigm to examine the mental imagery effects on processing prepositional phrases (PPs) encoding spatial (e.g., *in the drawer*) and abstract meanings (*in the fear*). The spatial meaning of the target preposition represents the prototypical sense, while the selected abstract meaning was chained to the prototypical spatial meaning and motivated by the conceptual metaphor (i.e., STATE IS A CONTAINER). In the semantic priming task, participants saw a related or unrelated schematic diagram prime embedded with a trajector (TR) word (e.g., *knife*) and then judged the grammaticality of the target PP containing a preposition and landmark^1^ (LM). Results showed compatibility effects on processing both spatial and abstract language in L2 adolescent English learners (Wang and Zhao, 2023) and interference effects on processing both spatial and abstract language in L2 adult English learners (Wang and Zhao, 2024). The existing evidence of interference and compatibility effects and their interactions with L2 proficiency is insufficient to conclude the patterns of L2 mental imagery in abstract language processing, hence further research on this issue is indispensable.

It was suggested that language proficiency is a significant factor influencing L2 mental imagery effects. Ahn and Jiang (2018) compared L1 and L2 mental imagery related to orientation and shape using the SPVT. Results indicated that both Korean NSs and advanced L2 Korean learners exhibited faster responses in the matching condition compared to the mismatching condition, suggesting native-like semantic integration abilities in advanced L2 proficiency. However, Chen et al. (2019) found distinctive patterns between L1 Cantonese, L2 Mandarin, and L3 English in SPVT results, with compatibility effects observed in L1 processing but no effects in L2 or L3, despite comparable proficiency levels in L1 and L2 but higher proficiency levels in L2 than L3. The results suggest robust evidence of L1 mental imagery but a conspicuous absence of embodied imagery in non-native language comprehension, implying distinct conceptual systems between L1, L2, and L3. Similarly, Norman and Peleg (2022) observed contrastive findings between L1 and L2 mental imagery. Using the SPVT, they investigated bilingual speakers’ L1-Hebrew and L2-English mental imagery effects of shape. Results showed compatibility effects in L1 processing, whereas this pattern was not observed in L2 processing with an intermediate level, leading the authors to argue for reduced mental imagery effects in L2 relative to L1.

Two possible accounts can explain the interactions between L2 proficiency and mental imagery. Firstly, limited L2 proficiency can result in considerable cognitive resources allocated to L2 comprehension, leaving fewer resources for perceptual simulation (Atkinson, 2010). This often leads to partial simulation (Norman and Peleg, 2022) or even no simulation (Chen et al., 2019). Secondly, compared to L1, there is a weaker link between perceptual representations and L2, as L2 comprehension may not be as grounded in sensorimotor knowledge as L1 comprehension (Dudschig et al., 2014). This discrepancy leads to distinct formations of L1 and L2 mental representations, resulting in different mental imagery outcomes in L1 and L2 (Chen et al., 2019; Norman and Peleg, 2022). However, as L2 proficiency increases, L2 mental representations may converge with the established L1 representation system (Foroni, 2015), potentially reducing differences between L1 and L2 imagery (Ahn and Jiang, 2018).

Moreover, L2 mental imagery may be influenced by the context of language acquisition. Participants in these studies were late bilinguals who acquired L1 in naturalistic settings and received L2 instruction primarily in formal school settings (Ahn and Jiang, 2018; Chen et al., 2019; Norman and Peleg, 2022). Due to different contexts of language acquisition, the sensorimotor activation in L1 and L2 can be distinct. For late bilinguals acquiring L2 after puberty, their perceptual systems have been shaped by the fully developed L1 system (Pavlenko, 2005; Perani and Abutalebi, 2005; Dudschig et al., 2014). However, with accumulated exposure to L2 instruction and increased L2 proficiency, weaker connections between perceptual representations in sensorimotor neurons and L2 can become stronger and richer (Monaco et al., 2019). In summary, these divergent findings related to proficiency and the context of language acquisition underscore the need for further investigation of their interaction with L2 mental imagery.

Building upon evidence from empirical L2 mental imagery studies and the theoretical model of simulation-based L1 comprehension (Bergen and Chang, 2005, 2013), we propose a simulation-based L2 comprehension model. We hypothesize that the L2 model shares three primary processes—*constructional analysis*, *contextual resolution*. and *embodied simulation*—with slight variations in moderators compared to the L1 model. We posit that L2 mental imagery can be influenced by language-internal, learner, and contextual factors. Firstly, the identification of L2 constructions, based on both L2 forms and meanings, can be influenced by corresponding elements in L1. The language-internal factors, known as the L1 transfer (Ortega, 2013) or L1 entrenchment (MacWhinney, 2005), may have positive or negative effects depending on cross-linguistic similarities and differences. Learner factors such as L2 proficiency might impact the *constructional analysis* process. Similar to the L1 model, semantic specifications are identified during *contextual resolution* and then resolved for *embodied simulation* in the L2 model. Throughout these processes, world knowledge and communicative context are incorporated as contextual factors, instantiated by the length of immersion in an L2 environment and the amount of communication in the L2. Notably, we emphasize the role of the instructional context quantified by the amount of L2 classroom instruction. The context of acquisition is assumed to be a key differentiating factor that may impact the mental imagery effects between L1 and L2 comprehension. Finally, after the simulation process, contextually appropriate inferences are generated to support L2 comprehension.

## 2 The present study

Theoretically, we aim to validate the proposed L2 mental simulation model by examining language-internal, learner, and contextual factors. Existing studies have discussed potential influential factors of L2 mental simulation, with relatively more studies focusing on language-internal (Vukovic and Williams, 2014; Tomczak and Ewert, 2015; Wu, 2016; Koster et al., 2018) and learner factors (Wheeler and Stojanovic, 2006; Qian, 2016; Ahlberg et al., 2018; Ahn and Jiang, 2018; Chen et al., 2019) and less attention on contextual factors (Ahn and Jiang, 2018; Chen et al., 2019; Norman and Peleg, 2022). Given the limited quantitative testing of the contextual factor in previous studies, the current study aims to explore its contribution to mental simulation effects in L2 processing.

The present study examined mental imagery of spatial directionality in two satellite languages, Mandarin Chinese and English. In Mandarin, directionality is typically encoded in a resultative verb compound (RVC) construction comprised of two components: a displacement verb and a directional verb (Li and Thompson, 2009). The displacement signals the manner of motion (e.g., *zǒu*, ‘walk’, and *pǎo,* ‘run’) or changes in conditions or situations (e.g., *tuī*, ‘push’, and *sòng*, ‘send’). The directional verb (e.g., *jìn*, ‘enter’, and *chū,* ‘exit’) indicates the path of motion or the directional result of the action implied by the displacement verb. In English, manner is typically encoded in verbs, and path is encoded in prepositions. *Into* and *out of* as translation equivalents of Chinese directional verbs *jìn* (‘enter’) and *chū* (‘exit’) express dynamic paths of motion deriving from the non-dynamic prepositions *in* and *out* (Li and Thompson, 2009; Lindstromberg, 2010). The spatial sense of *into* expresses “a spatial relation in which the TR is located on the exterior of a bounded LM and is oriented toward the LM” (Tyler and Evans 2003, pp. 199). Tyler and Evans (2003) argued a parallel distinction between *out* and *out of* and between *in* and *into.* Therefore, the spatial sense of *out of* expresses a spatial relation in which the TR is located on the interior of a bounded LM and is oriented away from the LM. An abstract sense is also selected according to the conceptual metaphor STATE IS A CONTAINER for each English preposition (Lakoff and Johnson, 1980; Lakoff, 1987) and for each Chinese directional verb (Yin, 2011). Table 1 presents the spatial and abstract meanings of two Chinese directional verbs (*jìn*, ‘enter’ and *chū,* ‘exit’) along with two corresponding diagrams with sample sentences. These similarities allow cross-linguistic comparisons between the two languages.

**Table 1:** Diagrams, senses and sample sentences for *jìn* (enter) and *chū* (exit).

Target	Diagram	Sense	Sample sentences				
*jìn*	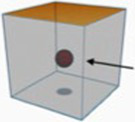	Spatial	一些 *yì-xiē* some	游客 *yóu-kè* tourist	走 *zǒu* walk	进 *jìn* enter	剬园。 *gōng-yuán* park
			‘Some tourists walked into the park.’				
		Abstract	一些 *yì-xiē* some	毕业生 *bì-yè-shēng* graduate	踏 *tà* step	进 *jìn* enter	社会。 *shè-huì* society
			‘Some graduates stepped into society.’				
*chū*	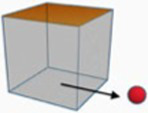	Spatial	一些 *yì-xiē* some	职员 *zhí-yuán* staff	走 *zǒu* walk	出 *chū* exit	办剬室。 *bàn-gōng-shì* office
			‘Some staff walked out of the office.’				
		Abstract	一些 *yì-xiē* some	艺术家 *yì-shù-jiā* artist	淡 *dàn* fade	出 *chū* exit	圈子。 *quān-zi* circle
			‘Some artists faded out of the circle.’				

Methodologically, our study applies an innovative approach by implementing a sentence-*diagram* verification task (SDVT), aiming to refine existing methods to address current limitations and provide a more nuanced understanding of mental imagery processes in both L1 and L2 contexts. These diagrams, three-dimensional image schematic representations, capture the spatial configurations of both concrete and abstract meanings in language (Richardson et al., 2003; Tyler and Evans, 2003; Langacker, 2008) and illustrate visual contrasts and figure-ground relationships in mental configurations. Furthermore, diagrams play a crucial role in studying mental abstraction, which demands a higher level of imagination (Zwaan, 2014). In contrast, the SPVT paradigm used in prior sentence-processing studies with concrete pictures (Stanfield and Zwaan, 2001; Zwaan et al., 2002; de Koning et al., 2017b; Schütt et al., 2023) captures the lowest level of embeddedness (i.e., demonstration) and falls short in providing reliable imagery cues for abstract mental concepts and measuring mental representations of abstract language meanings accurately. Schematic diagrams, being abstract visual symbols, are more suitable than pictures for investigating mental representations triggered by the processing of abstract grammatical and semantic domains such as tense-aspect-modality (Tyler et al., 2010; Tyler and Jan, 2017), countability (Langacker, 2008), and figurativeness (Holme, 2004). These domains are argued to have theoretical underpinnings in concrete spatial domains (Lakoff, 1987).

In summary, further empirical evidence is required to substantiate and refine the proposed L2 mental imagery model. Due to the limited research on L2 mental imagery, particularly the scarcity of L2 studies utilizing schematic diagrams to investigate mental imagery in bilingual language processing Wang and Zhao, (2023, 2024), it remains challenging to generalize the extent to which L2 aligns with or diverges from L1 mental imagery and the factors influencing these differences. Motivated by these research gaps, the present study employs an innovative SDVT to explore the presence of perceptual representations or mental imagery during language comprehension in adult L1 Chinese (Experiment 1) and L2 English sentence processing (Experiment 2). Guided by the simulation-based L2 understanding model, we manipulate two semantic specifications, namely spatial directionality and abstractness of senses. More specifically, the study aims to address the following research questions:

1. Do Chinese L2 learners of English enact mental imagery in L1 Chinese and L2 English sentence processing?
2. If yes, to what extent is the mental imagery modulated by spatial directionality (*jìn / into* vs. *chū / out of*) and abstractness of senses (spatial vs. abstract) in L1 Chinese and L2 English, respectively?
3. Does contextual factor interact with L2 mental imagery?

## 3 Experiment 1

### 3.1 Participants

21 Chinese adults (4 males and 17 females) were recruited from a public university in Australia (mean age = 22.62, *SD* = 1.94). Among them, 9 were undergraduates and 12 were postgraduates majoring in fields such as arts, education, science, and commerce. All participants spoke Mandarin Chinese as their L1. They were asked to rate their L1 proficiency on a numeric scale ranging from 10 to 100^2^, and their average self-rated L1 proficiency was 89.05 (*SD* = 13.48). Additionally, some participants reported knowledge of other languages, including Cantonese (*n* = 2), Japanese (*n* = 2), Korean (*n* = 1), and German (*n* = 1). Moreover, several participants were proficient in various Chinese dialects, including Shanghainese (*n* = 2), Wu dialect (*n* = 2), Anhui dialect (*n* = 1), Fujian dialect (*n* = 1), Hebei dialect (*n* = 1), Sichuan dialect (*n* = 1), Zhoushan dialect (*n* = 1) and Suzhou dialect (*n* = 1). Informed written consent was obtained from each participant in advance. Upon task completion, each participant received monetary compensation for their time of participation.

### 3.2 Materials and design

Experiment 1 aimed to test whether the shared image schemas between spatial and abstract senses could generate mental imagery effects in L1 Chinese sentence processing. The stimuli in Experiment 1 consisted of 80 target sentences (20 sentences × 2 directional verbs × 2 senses) and 40 filler sentences. Among the 80 target sentences, 56 sentences (14 sentences × 2 directional verbs × 2 senses) were used as the SDVT stimuli, and the remaining 24 sentences (6 sentences × 2 directional verbs × 2 senses) were used as the semantic rating task stimuli. For the SDVT, we adopted a 2 directional verb (*jìn*, *chū*) × 2 sense (spatial, abstract) × 2 Congruency conditions (matching, mismatching) factorial Latin-square design. To counterbalance the target sentence stimuli in the matching and mismatching conditions, we created two stimuli lists so that there was no overlap of target sentence stimuli between the matching and mismatching conditions. In addition to the 56 target sentences, each SDVT stimuli list comprised 40 filler sentences, which remained the same in the two counterbalanced lists. All filler sentences were adapted from sample sentences in the Chinese grammar book (Ross and Ma, 2014), which had comparable lengths to the target sentences but did not involve the two target Chinese directional verbs (e.g., *shí táng de yān cōng yī dào zhōng wǔ jiù mào yān*, ‘The canteen chimney starts to emit smoke at noon’). In addition to the target *into* and *out-of* diagrams, two diagrams representing the UP-DOWN schema were created as fillers in the SDVT. Altogether, 96 sentences and 4 diagrams were used in the SDVT. For the semantic rating task, there was only one stimuli list with 24 target sentences but no filler sentences. The target sentences in the SDVT and semantic rating task shared the same syntactic construction with six segments, including a determiner, an adjective, a subject, a displacement verb, a directional verb, and an object noun (Example 1). There was no overlap in the target sentence stimuli between the two tasks (Supplementary material 1).

**Example 1**


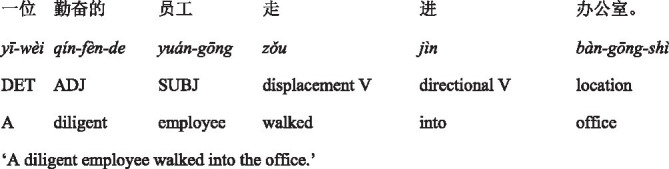


Frequencies of RVC phrases and RVC-object collocations in 80 target sentences were checked using the Corpus of Chinese Linguistics (CCL) (Zhan et al., 2003, 2019). After log-transformation, one-way ANOVA results revealed no significant differences in RVC phrasal frequency between items of the two directional verbs (*F* = 1.808, *p* = 0.183) or senses (*F* = 3.396, *p* = 0.069). Similarly, there were no significant differences in RVC-location collocation frequency between items of the directional verbs (*F* = 1.104, *p* = 0.297) or senses (*F* = 0.302, *p* = 0.584). Additionally, the sentence lengths in characters between stimuli of directional verbs (*F* = 1.960, *p* = 0.165) or senses (*F* = 0.002, *p* = 0.962) were balanced.

**Figure 1:**
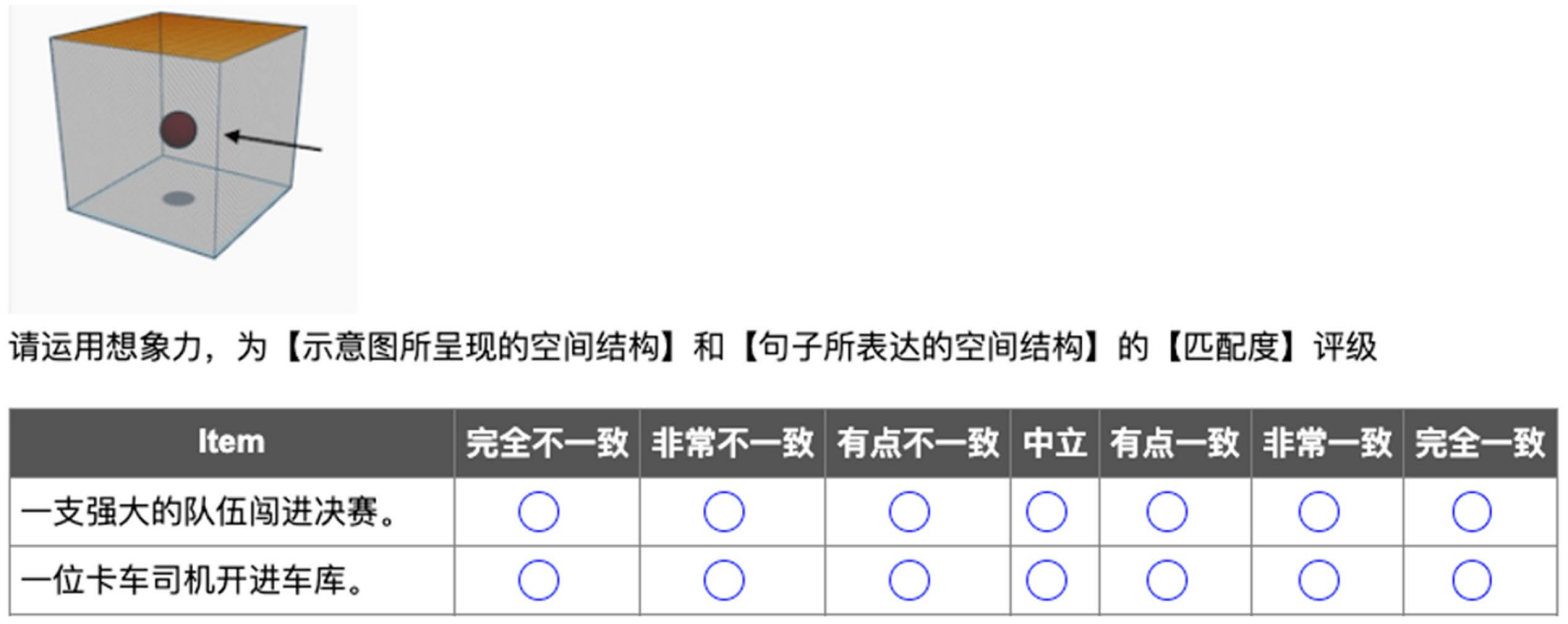
Sample stimuli of the Chinese semantic rating task.

To norm the semantic congruency between the conceptualizations of embodied scenes in two diagrams and Chinese sentences containing two directional verbs, an untimed semantic rating task was conducted. In this task, participants were presented with two blocks one by one. In each block, they saw one of the two diagrams (*into* or *out-of* diagram) and 12 Chinese sentences containing the corresponding directional verbs *jìn* (‘enter’) or *chū* (‘exit’). Half of the sentences expressed the spatial meaning, while the other half expressed the abstract meaning. Participants were instructed to rate the consistency of spatial configurations between diagrams and Chinese sentences on a 7-point Likert scale (1 *= completely inconsistent,* 7 *= completely consistent*) (see Figure 1). No time constraints were imposed, and no corrective feedback was provided during this task.

**Figure 2:**
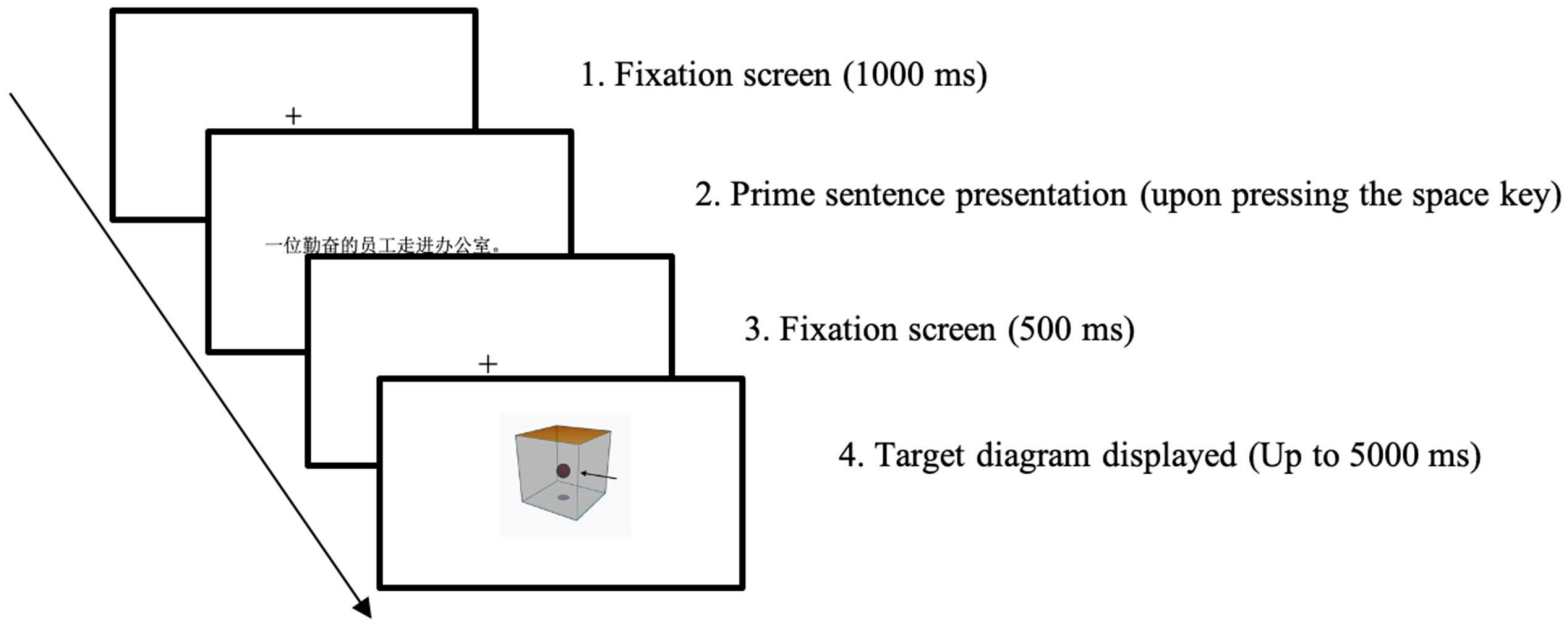
A sample matching trial of the SDVT (prime—a sentence of spatial sense of *jìn*; target—*into* diagram).

The SDVT in the current study followed the Chinese SPVT procedure described by Chen et al. (2020). Participants were initially presented with a fixation spot for 1,000 milliseconds. Subsequently, a prime sentence was displayed at the center of the screen (e.g., *yī-wèi qín-fèn-de yuán-gōng zǒu jìn bàn-gōng-shì*, translated as ‘A diligent employee walked into the office.’). Participants read the prime sentence at their own pace and pressed the space bar as soon as they finished reading. Once the space bar was pressed, the prime sentence was replaced by another fixation point at the center of the screen, which remained visible for 500 milliseconds. Finally, participants were presented with a diagram and tasked with verifying whether the spatial configuration depicted in the diagram was consistent with the meaning conveyed in the sentence they read. Participants made a binary judgment within 5 s by pressing ‘F’ or ‘J’ on the keyboard, representing ‘No’ or ‘Yes’ responses (Zwaan et al., 2004). If a response was not made within 5 s, the screen advanced to the fixation point for the next trial. A sample trial, depicting a sentence containing the spatial sense of *jìn* in the matching condition, is illustrated in Figure 2.

To familiarize participants with the SDVT procedure, a practice session was added before the formal session. In the practice phase, participants completed 20 practice trials and received corrective feedback with L1 explanations on each practice trial. The explanations demonstrate the one-on-one corresponding relationship between the TR and LM in the sentence stimuli and their referents (the red circle and gray cube) in diagram^3^. Data from practice trials were excluded from the analysis. In the formal session, participants did 96 trials without any feedback. Only the RTs (from the onset of the diagram display to the onset of a button response) and ARs of the trials in the formal session were analyzed.

### 3.3 Procedure

Data collection sessions were implemented online using PsyToolkit (version 3.4.4)(Stoet, 2010, 2017). Before the commencement of the experiment, written informed consent was obtained from each participant. Following this, participants completed a demographic questionnaire, which gathered basic information including gender, age, educational background, and language history. Subsequently, participants were randomly assigned to one of the two counterbalanced lists and completed the SDVT. After a short break, the untimed semantic rating task was carried out. The reason for conducting the Chinese semantic rating task after the SDVT was to minimize the potential influence of revealing the research focus through the rating task before the SDVT. Each data collection session had a duration of approximately 20 min. Only one attempt was allowed for each participant to complete the tasks, and they were not permitted to revisit or modify their previous answers.

### 3.4 Data analysis

Data were analyzed using *R* software (version 4.0.3) (R Core Team, 2024). Before the analysis, data trimming was performed. Since all participants achieved ARs above 80% (ranging from 89 to 100%) in the SDVT, all participants’ data were deemed reliable and included in the data pool. Only RTs with correct diagram verification responses to target trials in the formal task phase were subjected to analysis. Trials with verification RTs shorter than 200 milliseconds and longer than 3,000 milliseconds were excluded due to unreliability, resulting in the removal of 2.6% of data points. The lme4 package (Bates et al., 2022) was used to construct mixed-effects models, which tested the fixed effects of the condition and the random effects of participants and stimuli on RTs. The lmerTest package (Kuznetsova et al., 2017) was used to calculate *p* values. Semantic ratings, RTs, self-rated L1 proficiency, and sentence reading time were log-transformed. RTs were analyzed using linear mixed-effects models (Linck and Cunnings, 2015). We included random intercepts for participants and items and by-participant random slopes for directional verbs and senses. Self-rated L1 proficiency and sentence reading time were treated as covariates in the initial model. We used anova function to compare the fits of models and justify the choice of these models. The models converged well and were checked for statistical assumptions. The emmeans package (Lenth et al., 2023) was used to apply Tukey correction for pairwise comparisons. Cohen (1977) was reported as the effect size for RTs and was interpreted based on the recommendation in Plonsky and Oswald (2014): 0.60, 1.00, 1.40 corresponding to small, medium, and large effect sizes for within-subject contrasts, and 0.40, 0.70, and 1.00 as small, medium, and large effect sizes for between-group contrasts. Graphics were generated using the ggplot2 package (Wickham, 2016).

### 3.5 Results

#### 3.5.1 Results of the Chinese semantic rating task

Table 2 presents the means and SDs of semantic ratings for the consistency between diagrams and sentences involving two directional words with spatial and abstract senses. The results of one-way ANOVA revealed no significant differences in the ratings across the four diagram—sense categories (*F* = 1.187, *p* = 0.32), between directional verbs (*F* = 1.248, *p* = 0.271), or between senses (*F* = 2.075, *p* = 0.157). Given that the average rating scores all exceeded 6 out of 7, it can be concluded that the diagrams were consistently and reliably aligned with the spatial configurations of both the spatial and abstract senses of the two Chinese directional words in the sentences. Consequently, responses verifying a matching diagram after reading a sentence with a consistent meaning were categorized as correct judgments, while responses rejecting a mismatching diagram after reading a sentence with an inconsistent meaning were classified as incorrect judgments in the SDVT.

**Table 2:** Descriptive statistics of the Chinese semantic rating task.

	*Into* diagram – *jìn* (‘enter’) sentences		*Out-of* diagram – *chū* (‘exit’) sentences	
	Spatial	Abstract	Spatial	Abstract
Mean (SD)	6.32 (1.26)	6.02 (1.37)	6.73 (0.36)	6.27 (0.91)

#### 3.5.2 Results of the L1-SDVT

Table 3 shows the means and SDs of sentence reading time, and RTs and ARs of diagram verification by Directional verb, Sense, and Congruency of the Chinese SDVT.

**Table 3:** Descriptive statistics of sentence RTs, and diagram RTs and ARs of the Chinese SDVT.

		*jìn* (‘enter’)				*chū* (‘exit’)			
		Spatial		Abstract		Spatial		Abstract	
		Mean	SD	Mean	SD	Mean	SD	Mean	SD
Sentence reading time (ms)	Matching	1664.5	1054.1	1647.1	1045.7	1452.5	1030.3	1603.8	1079.3
	Mismatching	1529.7	1384.2	1663.7	1261.0	1644.4	1167.0	1567.5	1086.7
Verification RTs (ms)	Matching	890.6	373.5	922.2	443.8	962.0	430.1	998.9	427.3
	Mismatching	1088.3	429.4	1151.8	538.7	1149.1	423.3	1254.4	492.1
Verification ARs (%)	Matching	100.0	0.0	98.6	11.6	93.2	25.3	94.6	22.8
	Mismatching	98.0	14.1	91.2	28.5	95.9	19.9	97.3	16.3

*ms, milliseconds.

We compared the fits of the three-way interaction model^4^ with the two-way interaction model^5^. The results showed the two-way interaction model better fit the data. Results of the two-way interaction model revealed that sentence reading time was a significant covariate, but L1-Mandarin self-rated proficiency was not. Sense did not have significant fixed effects on RTs or have significant interaction with Congruency (Supplementary material 2). After removing the non-significant covariate and Sense, results of the simplified model (Table 4) showed sentence reading time was a significant covariate, indicating as sentence reading time increased, RTs of diagram verification increased. Results also revealed that Directional verb and Congruency had significant fixed effects, but their interaction was not significant.

**Table 4:** Results of the linear mixed-effects model for RTs of the Chinese SDVT.

Fixed effects	*b*	SE	95% CI	*t*	*p*
Intercept	6.20	0.15	[5.91, 6.49]	41.76	< 0.001***
Log (Sentence reading time)	0.07	0.02	[0.03, 0.11]	3.75	< 0.001***
Congruency	0.23	0.03	[0.17, 0.28]	7.93	< 0.001***
Directional verb	0.09	0.04	[0.02, 0.17]	2.34	0.019*
Directional verb × Congruency	−0.02	0.04	[−0.10, 0.06]	−0.42	0.677

Random Effects			
	Variance	S.D.	Correlation
Participant (Intercept)	0.035	0.19	
Item (Intercept)	< 0.001	0.02	
Directional verb (slope)	0.015	0.12	−0.53
Residual	0.112	0.34	

Model fit		
*R* ^2^	Marginal	Conditional
	0.10	0.29
Model equation: log(RT) ~ log(Sentence reading time) + Directional verb * Congruency + (1 + Directional verb | participant) + (1 | stimuli)		

***p < 0.001, **0.001 < *p* < 0.01, *0.01 < *p* < 0.05.

**Figure 3:**
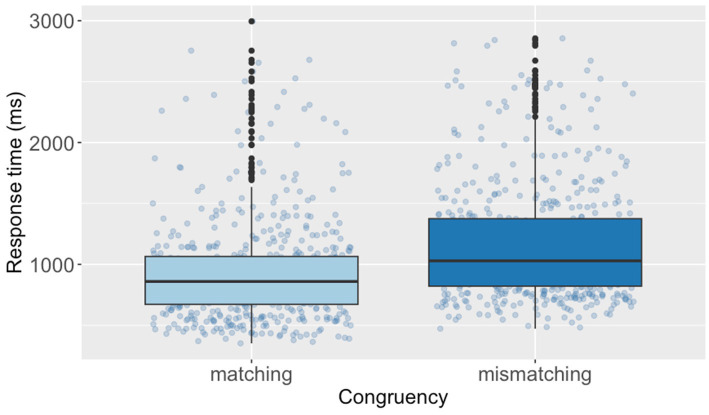
Response times of diagram verification by congruency of the Chinese SDVT.

**Figure 4:**
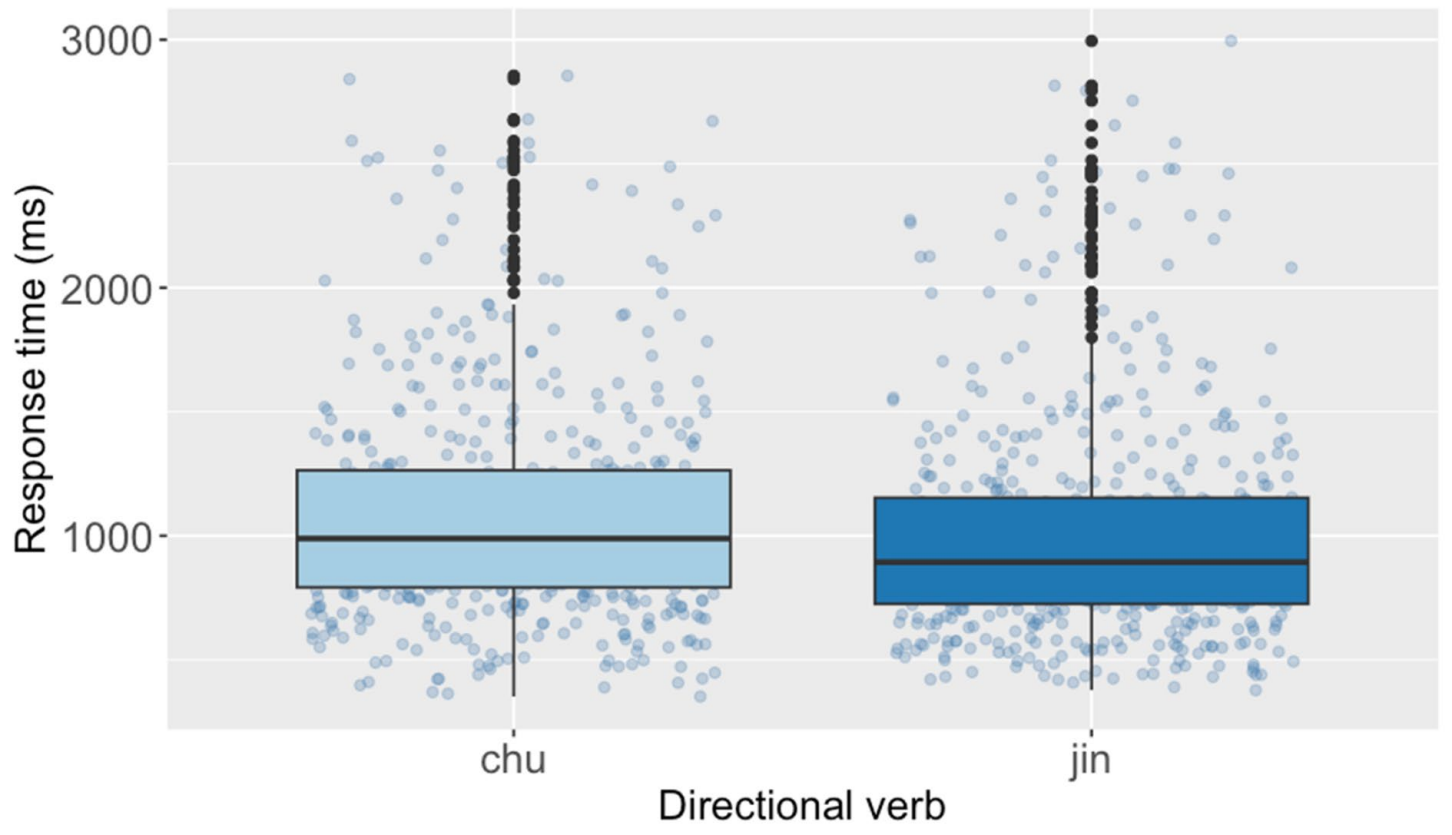
Response times of diagram verification by directional verb of the Chinese SDVT.

The *post hoc* analyses revealed that the mean RTs in the matching condition [*M* = 883, *SE* = 34.0, *df* = 23.6, 95% CI (816, 957)] were estimated to be 214 ms shorter than those in the mismatching condition [*M* = 1,097, *SE* = 42.4, *df* = 23.8, 95% CI (1,013, 1,188)] [Cohen’s *d* = 0.65, *SE* = 0.06, *df* = 23.6, 95% CI (0.52, 0.78), corresponding to a small compatibility effect]. Furthermore, the *post hoc* analyses indicated that the mean verification RTs after reading sentences with *jìn* (‘enter’) [*M* = 944, *SE* = 41.1, *df* = 20.5, 95% CI (862, 1,033)] were estimated to be 83 ms shorter than those of *chū* (‘exit’) [*M* = 1,027, *SE* = 39.3, *df* = 20.7, 95% CI (948, 1,112)] [Cohen’s *d* = 0.25, *SE* = 0.10, *df* = 20.5, 95% CI (0.04, 0.47), corresponding to a small effect]. Figures 3, 4 present the RTs of diagram verification by Congruency and Directional verbs, respectively. Additionally, we build a follow-up model^6^ including self-rated proficiency as a covariate to examine the sentence reading time (Supplementary material). Results revealed no significant fixed effects of any variables.

## 4 Experiment 2

### 4.1 Participants

20 adult L1-Chinese learners of L2-English (3 males and 17 females) were recruited from a public university in Australia (Mean age = 24.60, *SD* = 3.91). All participants were postgraduates pursuing a master’s degree in applied linguistics. Their average onset age of English learning was 8.60 (*SD* = 3.19) years old. On average, they spent 9.90 h per week reading English articles (*SD* = 8.39). The length of study abroad experiences ranged from 1 to 50 months (Mean = 15.00, *SD* = 15.33).

According to the Common European Framework of Reference (CEFR, Council of Europe, 2020), all participants were classified as higher intermediate to advanced L2 learners since their overall IELTS score fell between 6.5 and 7.5, with no bands less than 6.0 (Mean = 6.80, *SD* = 0.30). Their IELTS reading score ranged from 6.5 to 8.5 (Mean = 7.13, *SD* = 0.60). In addition to English, most participants reported some knowledge of other languages, including Japanese (*n* = 5), French (*n* = 3), Korean (*n* = 2), Cantonese (*n* = 1), German (*n* = 1), Thai (*n* = 1) and Latin (*n* = 1). Furthermore, many participants were also proficient in various Chinese dialects, such as Teochew dialect (*n* = 3), Hokkien (*n* = 2), Hunan dialect (*n* = 1) and Henan dialect (*n* = 1). Upon task completion, each participant received monetary compensation for their time. No participant in Experiment 1 participated in Experiment 2.

### 4.2 Materials and design

Experiment 2 aimed to investigate the mental imagery effects in L2-English online sentence processing. A timed SDVT in English was conducted by adopting the same factorial Latin-square design as Experiment 1. The same untimed semantic rating task was conducted in English to check the semantic consistency between the conceptualizations of the embodied scenes represented by the diagrams and the English sentences containing prepositions.

Experiment 2 utilized the same diagrams as Experiment 1 and targeted both spatial and abstract senses of English prepositions *into* and *out of*. All 80 target sentence stimuli in Experiment 2 were translation equivalents of Chinese sentence stimuli used in Experiment 1 (e.g., *A diligent employee walked into the office*). The stimuli include 56 target sentences and 40 filler sentences for the English SDVT (2 lists), and 24 target sentences for the semantic rating task. All target sentences were generated by following the sentence structure of *determiner + adjective + noun + verb + preposition + determiner + noun*. The frequencies of verb – preposition collocations and verb – preposition – location collocations were checked using the Corpus of Contemporary American English (COCA) (Davies, 2008). After log-transformation, the results of one-way ANOVA indicated no significant differences in the verb – preposition collocation frequency between prepositions (*F* = 2.504, *p* = 0.118) or senses (*F* = 2.710, *p* = 0.104). Similarly, no significant differences were observed in the verb – preposition – location collocation frequency between prepositions (*F* = 0.003, *p* = 0.953) or senses (*F* = 1.459, *p* = 0.231), as well as in the sentence length of characters between prepositions (*F* = 0.091, *p* = 0.764) or senses (*F* = 1.044, *p* = 0.310).

### 4.3 Procedure

The procedure of Experiment 2 was the same as Experiment 1.

### 4.4 Data analysis

Data were analyzed using *R* software (version 4.4.0) (R Core Team, 2024). Data trimming was conducted before the data analysis, following the same trimming criteria on the L1-SDVT data. Since all participants achieved ARs above 80% (ranging from 82 to 100%) in the English SDVT, data from all participants were deemed reliable and retained in the data pool. Only the RTs from target trials with the correct judgment responses in the formal task phase were analyzed. The trials in which the RTs were shorter than 200 milliseconds and longer than 3,000 milliseconds were excluded due to unreliability, resulting in the removal of 3.2% of data points. Experiment 2 used the same *R* packages and models to analyze the diagram verification RTs as Experiment 1. Variables of individual differences, including the age of acquisition, months of study abroad, hours of reading English articles, IELTS overall score, IELTS reading score, and sentence reading time were log-transformed and treated as covariates in the initial model.

### 4.5 Results

#### 4.5.1 Results of the English semantic rating task

Table 5 displays the means and SDs of semantic ratings for the consistency between diagrams and English sentences involving two prepositions with spatial and abstract senses. The results of one-way ANOVA revealed significant differences in the consistency ratings between the four diagram – sense categories (*F* = 9.276, *p* < 0.001). Tukey post-hoc analysis results indicated ratings to the spatial sense were significantly higher than the abstract sense, applying to both the *into* diagram (*p* = 0.031) and *out-of* diagram (*p* < 0.001).

**Table 5:** Descriptive statistics of English semantic rating task.

	*Into* diagram – *into* sentences		*Out-of* diagram – *out-of* sentences	
	Spatial	Abstract	Spatial	Abstract
Mean (SD)	6.44 (0.55)	5.74 (0.73)	6.54 (0.59)	5.41 (1.14)

#### 4.5.2 Results of the L2-SDVT

First of all, descriptive statistical analyses were conducted. Table 6 presents the mean and standard deviations of RTs of sentence reading, and RTs and ARs of diagram verification by Directional verb, Sense, and Congruency of the L2 English SDVT.

**Table 6:** Descriptive statistics of sentence RTs, and diagram RTs and ARs of the English SDVT.

		*Into*				*Out of*			
		Spatial		Abstract		Spatial		Abstract	
		Mean	SD	Mean	SD	Mean	SD	Mean	SD
Sentence reading time (ms)	Matching	2712.7	1615.9	3227.7	1876.1	3040.3	2026.5	3294.9	2410.3
	Mismatching	2695.2	1595.8	3239.0	2806.1	2709.3	1458.2	3510.5	2776.8
Verification RTs (ms)	Matching	1067.4	495.8	1122.3	485.1	1072.2	462.0	1111.4	451.5
	Mismatching	981.4	459.5	1141.4	532.2	980.5	363.9	1141.5	443.8
Verification ARs (%)	Matching	97.9	14.5	92.9	25.8	94.3	23.3	94.3	23.3
	Mismatching	97.1	16.7	87.1	33.6	94.3	23.3	95.0	21.9

*ms, milliseconds.

Results of the initial linear mixed-effects model^7^ showed no covariate except for the sentence reading time was significant. Neither Preposition nor its interaction with Congruency was significant (Supplementary material 2). After excluding the non-significant covariates and Preposition, the results of the simplified model revealed a significant covariate of sentence reading time, indicating as sentence reading time increased, RTs of diagram verification increased. Results also revealed significant fixed effects of Congruency and marginally significant interaction between Sense and Congruency but no significant fixed effects of Sense on verification RTs (Table 7).

**Table 7:** Results of the linear mixed-effects model for RTs of the English SDVT.

Fixed effects	*b*	SE	95% CI	*t*	*p*
Intercept	5.61	0.20	[5.22, 6.00]	28.50	< 0.001***
Log (Sentence reading time)	0.16	0.02	[0.12, 0.21]	6.79	< 0.001***
Congruency	−0.07	0.03	[−0.13, −0.01]	−2.29	0.022*
Sense	0.03	0.03	[−0.04, 0.09]	0.83	0.408
Sense × Congruency	0.08	0.04	[0.00, 0.17]	1.90	0.058.

Random effects			
	Variance	S.D.	Correlation
Participant (Intercept)	0.053	0.23	
Item (Intercept)	0.000	0.00	
Sense (slope)	< 0.001	0.02	−1.00
Residual	0.120	0.35	

Model fit		
*R*^2^	Marginal	Conditional
	0.07	0.34
Model equation: log(RT) ~ log(Sentence reading time) + Sense*relatedness + (1 + Sense | participant) + (1 | stimuli)		

***p < 0.001; **p < 0.01; *p < 0.05; *p* < 0.0.05.

**Figure 5:**
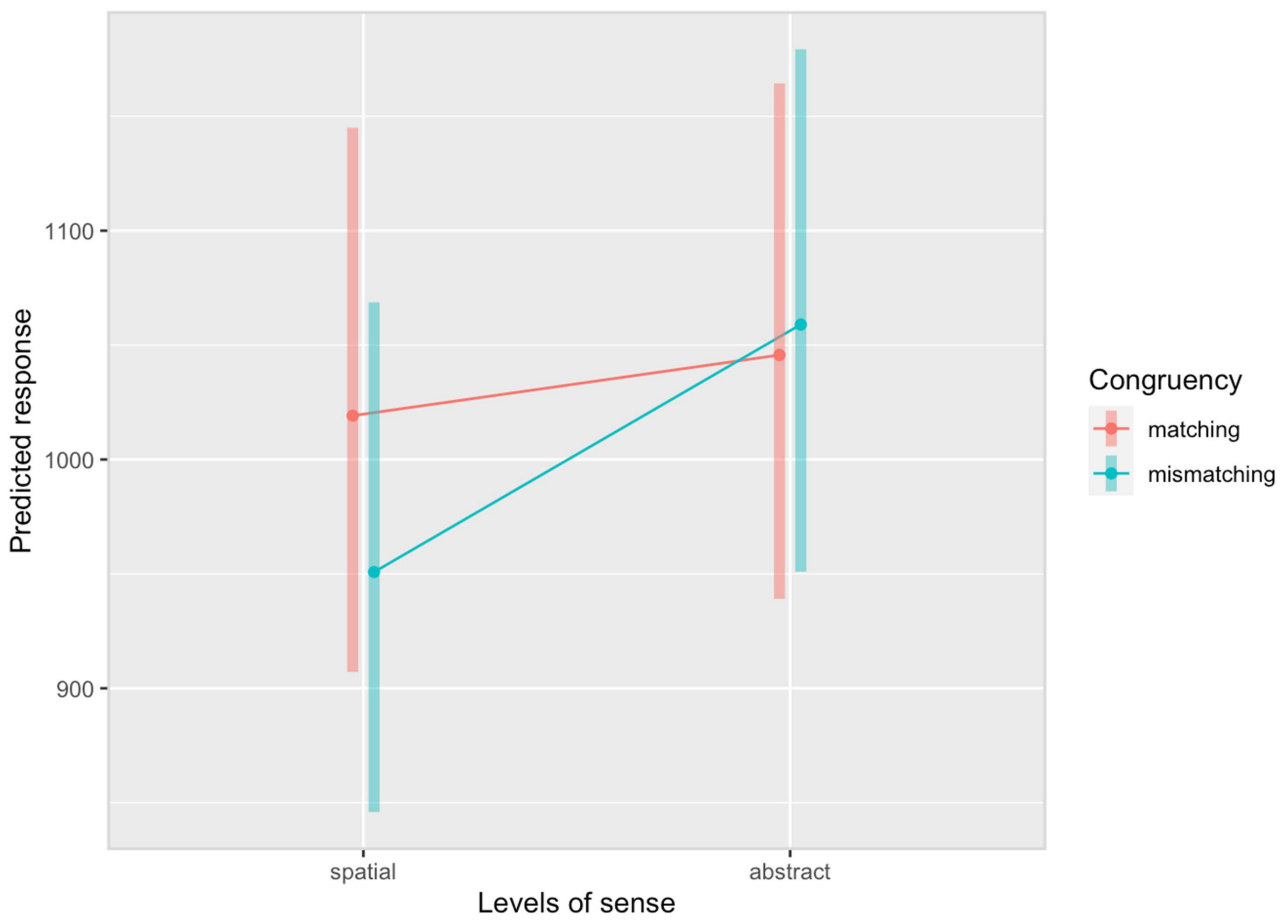
Response times of diagram verification by sense and congruency in the English SDVT.

The *post hoc* analysis results revealed the mean RTs in the matching condition [*M* = 1,032, *SE* = 53.5, *df* = 20.8, 95% CI (911, 1,170)] were estimated to be 29 ms longer than those in the mismatching condition [*M* = 1,003, *SE* = 52.1, *df* = 21.0, 95% CI (886, 1,137)], but this difference was not significant (*t* = 1.309, *p =* 0.196). Furthermore, *post hoc* analyses of the interaction between Sense and Congruency showed that the mean verification RTs after reading sentences encoding spatial senses in the matching condition [*M* = 1,019, *SE* = 57.2, *df* = 22.1, 95% CI (891, 1,166)] were estimated to be 68 ms longer than those in the mismatching condition [*M* = 951, *SE* = 53.7, *df* = 22.6, 95% CI (831, 1,088)] [Cohen’s *d* = 0.20, *SE* = 0.09, *df* = 22.1, 95% CI (0.02, 0.38), *t =* 2.289, *p* = 0.026, corresponding to a small interference effect]. Whereas the mean verification RTs after reading sentences encoding abstract senses between the matching condition [*M* = 1,046, *SE* = 54.3, *df* = 22.5, 95% CI (923, 1,184)] and the mismatching condition [*M* = 1,059, *SE* = 55.0, *df* = 22.6, 95% CI (935, 1,199)] were not significantly different (*t =* −0.408, *p* = 0.685). Figure 5 presents the RTs of diagram verification by Sense and Congruency.

Additionally, we built a separate model^8^ to examine the extent to which contextual factors and learner factors may interact with the L2 mental imagery process. Results revealed that the interaction between length of immersion and Congruency was significant [*b* = −0.04, *SE* = 0.02, 95% CI (−0.08, 0.00), *t =* −2.14, *p* = 0.033], the interaction between weekly hours of English communication and Congruency was marginally significant [*b* = 0.06, *SE* = 0.03, 95% CI (−0.01, 0.12), *t =* 1.80, *p* = 0.072], but neither the length of immersion (*t =* 0.86, *p* = 0.391) nor the weekly hours of English communication (*t =* 0.01, *p* = 0.991) itself had significant fixed effects. Post-hoc analysis did not show any significant results from these two interactions. Besides, sentence reading time itself had significant fixed effects [*b* = 0.21, *SE* = 0.03, 95% CI (0.14, 0.27), *t =* 6.47, *p* < 0.001], but its interaction with Congruency was not (*t =* −1.07, *p* = 0.285). The interactions between Congruency and learner factors, including the age of acquisition (*t* = −1.06, *p =* 0.290), IELTS overall score (*t* = −0.38, *p =* 0.701), and IELTS reading score (*t* = −1.00, *p =* 0.317), were not significant. None of these learner factors had significant fixed effects.

Finally, we built a follow-up model^9^ including all the learner and contextual factors as covariates to examine their impact on the L2 sentence reading time (Supplementary material 2). Results showed non-significant results of all covariates but a significant fixed effect of Sense [*b =* 0.14, *SE* = 0.05, 95% CI (0.04, 0.25), *t =* 2.65, *p* = 0.008]. After removing the non-significant covariates and Preposition variable, the *post hoc* analyses of the simplified model indicated the mean reading time of sentence encoding spatial senses [*M* = 2,367, *SE* = 211, *df* = 20.7, 95% CI (1909, 2,936)] was estimated to be 449 ms shorter than those encoding abstract senses [*M* = 2,816, *SE* = 270, *df* = 20.4, 95% CI (2,235, 3,548)] [Cohen’s *d* = 0.40, *SE* = 0.09, *df* = 20.4, 95% CI (0.21, 0.59), corresponding to a small effect].

## 5 General discussion

The present study applied an innovative SDVT paradigm to examine perceptual mental representations in both L1 Chinese and L2 English sentence comprehension. The results of the two experiments reveal distinct patterns of mental imagery in L1 and L2 processing. Experiment 1 demonstrates compatibility effects in L1 Chinese processing, where RTs of verifying diagrams in matching trials were faster than in mismatching trials. These compatibility effects were not modulated by Directional verb or Sense. Experiment 2 reveals interference effects in L2 English processing, where RTs of verifying diagrams in matching trials were slower than in mismatching trials. These interference effects were found to be modulated by Sense, which were observed after reading sentences encoding spatial senses but not abstract senses. Another difference between the SDVT results in the two experiments is that the Directional verb was found to modulate RTs of diagram verification after reading L1 Chinese sentences, with the RTs being faster after *jìn* (‘enter’) sentences compared to *chū* (‘exit’) sentences. However, Preposition did not modulate RTs of diagram verification after reading L2 English sentences. Contextual factors including the length of immersion and hours of English communication were found to interact with the L2 mental imagery process. This section first explains the mental imagery effects in L1 Chinese and L2 English processing, discusses the empirical evidence for the developed L2 mental imagery model, and concludes the study with current limitations and suggestions for future research.

### 5.1 Mental imagery effects in L1 and L2 sentence processing

#### 5.1.1 Mental imagery in L1 Chinese sentence processing

The compatibility effects observed in the current study align with previous mental imagery research that used picture stimuli in SPVTs and found similar compatibility effects in processing L1 Mandarin Chinese (Chen et al., 2020) and other languages such as English (Stanfield and Zwaan, 2001; Zwaan et al., 2002; Winter and Bergen, 2012) and Dutch (Chen et al., 2020; de Koning et al., 2017a, 2017b). The consistent compatibility effects extend the scope of L1 mental imagery measures from pictorial to diagrammatic visual representations. The overall compatibility effects suggest that when processing the sentence, the Chinese directional verbs (*jìn* and *chū*) encoding perceptual-motor meanings in the sentential context activate the CONTAINMENT schema and corresponding perceptual-motor neurons in the brain. When participants see a diagram whose spatial configuration is congruent with the perceptual-motor meanings expressed in the preceding sentences, the activated CONTAINMENT schema facilitates the visual processing of the diagram, leading to faster verification responses.

These findings support Bergen’s (2007) argument that compatibility effects are more likely to be observed when the sentence and visual stimuli are not temporally overlapped, and when the sentence stimuli precede visual stimuli. In the current experiment, the sentence and visual stimuli were presented sequentially. The sentence provides a linguistic context for the mental recreation of the embodied perceptual-motor experiences that are grounded in image schemas (Johnson, 1987; Lakoff, 1987; Gibbs, 2005). Additionally, participants read the sentence at their self-paced speed. The unlimited time for sentence reading promotes deep processing and comprehension of sentence meanings (Zwaan, 2014; Shaki and Fischer, 2023), enabling mental imagery to be enacted without time pressure.

Compatibility effects were observed in verifying both *into* and *out-of* diagrams. This finding may be attributed to the similarly high semantic consistency ratings between the two diagrams and the corresponding Chinese sentences containing the directional verbs *jìn* and *chū* in the semantic rating task. These ratings suggest both *into* and *out-of* diagrams serve as good visual representations of the spatial configurations expressed by the two directional verbs. However, the faster RTs for verifying the *into* diagram could be explained by the presence of an alternative directional verb *rù* (‘enter’), which shares the same meaning with *jìn* and can form RVCs with displacement verbs (e.g., *zǒu rù* ‘walk into’, *tà rù* ‘step into’, *fēi rù* ‘fly into’). *Rù* (‘enter’) also has a relatively high frequency of usage (543,848 instances for *rù*, 1,055,653 instances for *jìn* and 1,493,102 instances for *chū*) according to CCL (Zhan et al., 2003, 2019). In contrast, there is no alternative directional verb for *chū* in Mandarin Chinese. Consequently, the total linguistic instances expressing an “*into*” meaning were 106,399 more than instances expressing an “*out of*” meaning. This higher frequency of linguistic instances for *into* expressions suggests that Chinese speakers may encounter more situations of “being included by a bounded container” in daily life, such as “getting into a building” and “walking into an office,” compared to situations of “being excluded by a bounded container,” such as “leaving out of the country” and “stepping out of the comfort zone.” The richer embodied experiences lead to more exemplars of the spatial configurations depicted in the *into* diagram than the *out-of* diagram, resulting in a better understanding of the *into* configuration of the CONTAINMENT schema. Consequently, this supports intuitive verification judgments under time pressure and leads to faster RTs in verifying the *into* diagram compared to the *out-of* diagram.

The compatibility effects were found to apply to both spatial and abstract senses of Chinese directional verbs. It aligns with previous findings where associations between the orientation of image-schematic representations and the abstractness of verb meanings were identified (e.g., concrete: *lift*—vertical and *push*—horizontal; abstract: *hope*—vertical and *argue*—horizontal), demonstrating the consistency of image schema between concrete and abstract verbs (Richardson et al., 2001). In the current experiment, the spatial and abstract senses of Chinese directional verbs were grounded in the CONTAINMENT schema. Processing spatial and abstract senses could both activate the corresponding shared perceptual-motor neurons for the CONTAINMENT schema, leading to facilitation in the speed of processing congruent spatial configurations in the diagrams (Bergen, 2007). It supports the psychological reality of the embodied CONTAINMENT schema that underlies the literal and metaphorical spatial concepts (Gibbs, 2005; Spivey et al., 2005).

The compatibility effects observed in processing both spatial and abstract senses are consistent with the findings of the picture recognition task conducted by Richardson et al. (2003). In their study, English NS were asked to listen to a sentence (e.g., *The girl hopes for a pony*) and memorize pictures of the subject (e.g., *girl*) and object (e.g., *pony*). During the test phase, participants recognized pictures that were displaced either horizontally or vertically. English NSs’ RTs of recognizing picture pairs were faster when the picture display orientation matched the orientations implied in the verbs, with compatibility effects observed for both concrete and abstract verbs. These findings provide experimental evidence supporting the embodied nature of image schemas and suggest that image schema underlies the semantic association between literal and metaphorical senses in L1 (Lakoff, 1987; Gibbs, 1996).

#### 5.1.2 Mental imagery in L2 English sentence processing

In Experiment 2, longer RTs were identified in the matching condition compared to the mismatching condition when sentences encoded spatial but not abstract senses. The interference effect could be due to the simultaneous recruitment of the same sensorimotor neurons for processing linguistic and visual information (Bergen, 2007; Bergen et al., 2010). L2 learners tended to have difficulties integrating linguistic and visual information, especially when abstract meanings were conveyed Wang and Zhao, (2024). This causes a delay of RT in the matching condition relative to the mismatching condition of spatial sense only because it is more likely and intuitive for L2 English learners to map the LM (e.g., *office*) in a sentence that encodes a spatial sense onto the corresponding object in the diagram (i.e., the cube), as they both denote visible, tangible, and concrete objects. In contrast, it is less intuitive for them to map the LM in the target domain of a conventional metaphor (e.g., *society*) in a sentence that encodes an abstract sense because abstract concepts like *society* are often invisible and intangible, sharing less visual similarity with the diagrams. Mental representations of abstract concepts are more challenging to activate via diagrams, especially for those late L2 bilinguals whose mental associations between perceptual representations and L2 forms are weaker than L1 (Perani and Abutalebi, 2005; Dudschig et al., 2014; Monaco et al., 2019). The distinction of L2 mental imagery between spatial and abstract senses was also supported by higher ratings on the semantic consistency between the spatial sense and diagrams compared to the abstract sense in the L2 English semantic rating task. However, the semantic ratings for spatial and abstract sense were found to be similar in the L1 Mandarin semantic rating task. The discrepancy in abstract senses between L1 and L2 could be attributed to the fact that processing L1 and L2 figurative language was inherently different (Littlemore and Low, 2006; Littlemore et al., 2011; Shi et al., 2023), with the figurative language being more difficult for L2 learners to comprehend.

The L2 mental imagery effects modulated by spatial and abstract senses could stem from different cognitive mechanisms involved in processing literal and metaphorical languages, as evidenced by longer reading times for abstract senses compared to spatial senses (Shi et al., 2023). This account finds support in previous behavioral and ERP findings (Lai et al., 2009; Lai and Curran, 2013). According to the behavioral results in Lai et al. (2009), longer RT (about 110 ms) for processing conventional metaphors relative to literal language was observed. They compared the ERPs of processing literal and metaphorical sentences with different source-target domain mappings, wherein the sentence ended with the same target word (e.g., direction) but encoded a literal sense (e.g., ROAD-ROAD mappings in *The path turned in a new direction*) or a metaphorical sense (e.g., ROAD-LIFE mappings in *Her life has a new direction*). The amplitude of N400 effects was larger for the metaphorical sense than the literal sense (Lai et al., 2009; Lai and Curran, 2013), suggesting a higher cognitive load for processing conventional metaphors distinct from processing literal language.

### 5.2 L1 VS. L2 mental imagery and the proposed simulation-based L2 understanding model

Compared to the robust compatibility effects observed in L1 Chinese processing, the mental imagery effects in L2 English learners were largely attenuated, consistent with previous findings where compatibility effects were evident in L1 processing but reduced L2 mental imagery effects were observed (Foroni, 2015; Norman and Peleg, 2022). These findings support the argument that L2 comprehension may not be grounded in sensorimotor knowledge to the same extent as L1 comprehension (Dudschig et al., 2014). The reduced L2 effects also partially align with previous neurolinguistic results on L2 mental imagery of motion words, where less engagement of the motor cortex was found in processing L2 words compared to L1 words (Vukovic and Shtyrov, 2014). These findings further support the assumption that different cognitive mechanisms underlie L1 and L2 processing (Ullman, 2001).

One possible reason that might account for the discrepancy between L1 and L2 mental imagery effects in the current study is the contextual factor. This hypothesis finds support in the L2 results that the length of immersion and hours of English communication interacted with Congruency, suggesting the length of immersion in the L2 context and the amount of communication in L2 might potentially impact L2 mental imagery effects. Additionally, although all participants have some experience studying abroad, the length of living and studying in English-speaking countries varies widely, ranging from 1 to 50 months. Considering the L2 English learners’ average age of acquisition (8 years old), they are classified as late Chinese-English bilinguals who acquired Chinese in naturalistic settings but received English instructions mainly in formal school settings. Given that their L2 proficiency is at the higher intermediate to advanced level, they may not have encountered as many exemplars in L2 English as in L1 (Pavlenko, 2005; Perani and Abutalebi, 2005), potentially resulting in a weaker degree of embodiment in L2 (Semin and Smith, 2008; Foroni, 2015).

### 5.3 Limitations and future directions

**Figure 6:**
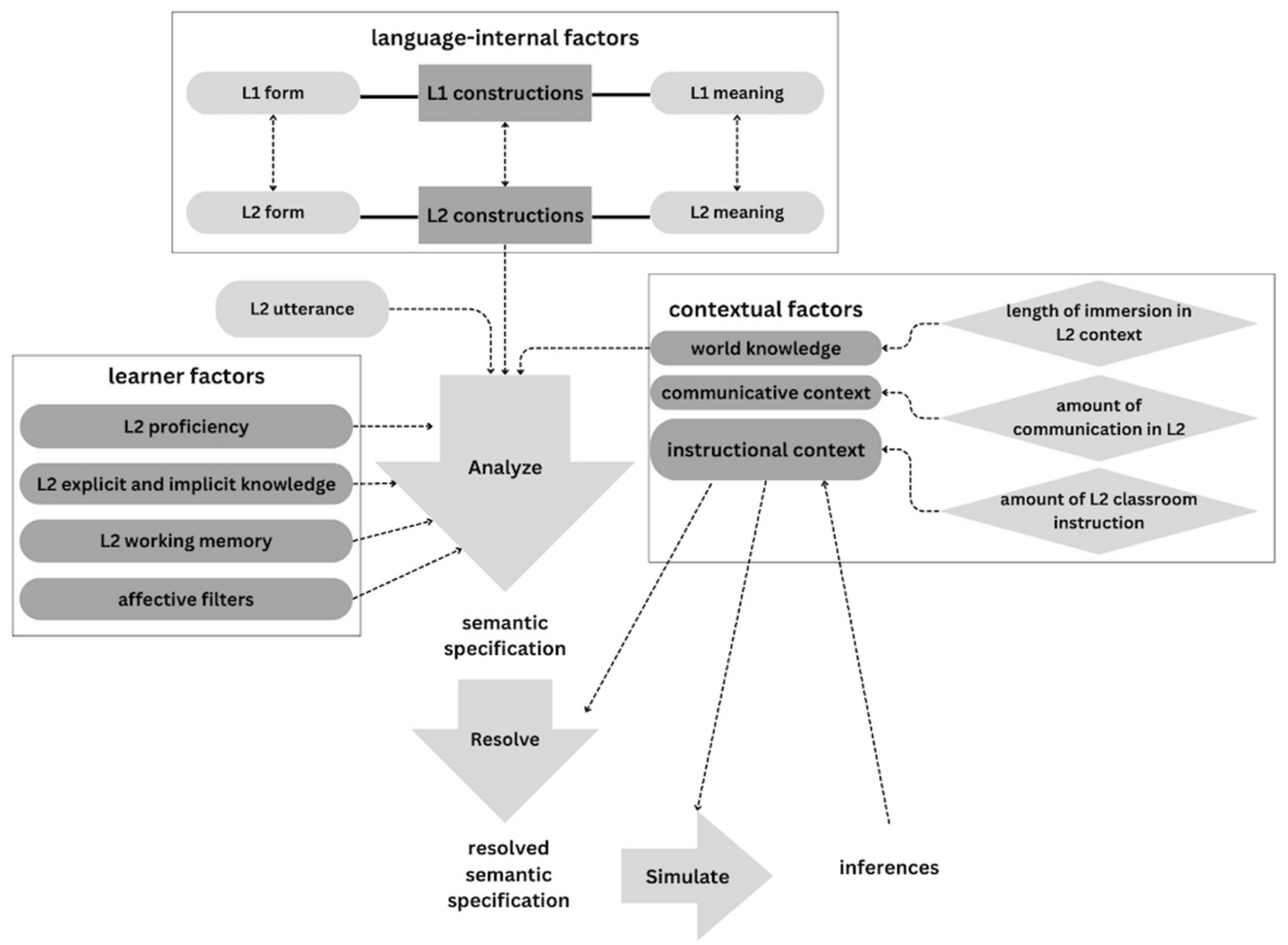
The developed simulation-based L2 comprehension model.

Due to the scope of the current study, we only considered L2 proficiency as the primary learner factor. Although we argued that L2 proficiency is a key factor contributing to the *constructional analysis* stage before the *embodied simulation* stage, we did not find a significant interaction between L2 proficiency and Congruency or a fixed effect of L2 proficiency on L2 English learners’ RTs of diagram verification. One potential reason could be that we operationalized L2 proficiency using the IELTS score, an ordinal variable with a limited range of variation. Another reason could be the 20 L2 learners recruited in Experiment 2 constituted a homogenous group, all studying the same major of a postgraduate degree, suggesting a similar level of L2 English proficiency. Therefore, future researchers may consider using other standardized English proficiency tests and replacing ordinal scales with numerical ones and further investigate the effect of L2 proficiency in the proposed simulation-based L2 comprehension model. Besides, other learner factors, such as explicit and implicit knowledge, working memory, and affective filters (e.g., motivation, attitude, anxiety, self-confidence, willingness to communicate, etc.), may also potentially influence the L2 mental simulation process. Future studies are encouraged to explore these factors with empirical evidence. Figure 6 illustrates the proposed simulation-based L2 comprehension model.

Secondly, given the L1 Mandarin participants in Experiment 1 are international students studying in Australia, there might be a potential impact of L2 on their L1 SDVT performance, which can be considered in future research by recruiting another group of adult Chinese native speakers who study at universities in China with less exposure to English outside classrooms and comparing their performances with the results in the current study. Meanwhile, future studies should also enlarge the sample size to benefit the statistical power of the models. In addition, since we did not aim to treat different types of Chinese displacement verbs as a research question, we did not manipulate the number of verb tokens in the sub-categories. Future research is recommended to investigate this research question by manipulating a balanced number between the sub-categories of (displacement) verbs. Furthermore, the current study compares Chinese-English bilinguals’ mental simulation in L1 Mandarin and L2 English. Future studies can address the same theoretical question by comparing L1 and L2 speakers’ mental simulation in the same target language.

Finally, another potential limitation of the current study is that the SDVT paradigm can only examine mental imagery at a terminal state, failing to capture the ongoing dynamics during the mental imagery process. Future studies could consider using a self-paced reading paradigm interleaved with diagrams to examine the dynamic process in mental imagery or combine the SDVT paradigm with time-course measurements (e.g., EEG and fMRI).

In conclusion, the current empirical validation of the simulation-based L2 understanding model and the innovative SDVT paradigm demonstrates that image schematic diagrams are valid tools for investigating the presence of perceptual representations resulting from both L1 and L2 sentence comprehension. The findings reveal a significant difference in accessing mental representations during L1 versus L2 sentence comprehension, with an overall compatibility effect in L1 processing (both spatial and abstract meanings) and an interference effect in L2 spatial-meaning processing. These findings align with the previous L2 mental imagery research using SPVTs, which has concluded an overall weaker mental imagery effect in L2 processing than in L1 processing. Contextual factors may also interact with the L2 mental imagery process. The current study supports the proposed simulation-based L2 understanding model and validates the SDVT paradigm in verifying bilinguals’ image schematic representations in spatial and abstract motion language processing. Future studies are encouraged to integrate this paradigm with time-course measurements to capture the dynamics in the mental imagery process and further test other learner factors in the proposed L2 model.

## Data Availability

The datasets presented in this study can be found in online repositories. The names of the repository/repositories and accession number(s) can be found in the article/Supplementary material.

## Footnotes

^1^TR and LM constitute a spatial relationship, in which TR is the focus while LM is the secondary focus (Langacker, 2008). TR refers to the conceptually movable object whose location is described and evaluated relative to the stationary LM (Herskovits, 1997).

^2^10 *=* extremely poor, 20 = very poor, 30 = poor, 40 = limited, 50 = average, 60 = standard, 70 = good, 80 = very good, 90 = excellent, 100 = native-like.

^3^For example, the English translation of the L1 explanation of the sample stimuli was “The red circle represents *yuán-gōng* (‘employee’) and the gray cube represents *bàn-gōng-shì* (‘office’). The red circle points to the inside of the gray cube, so the spatial configuration depicted in the diagram was consistent with the meaning conveyed in the sentence. You should press ‘J’.”

^4^Model equation: log(RT) ~ log(Proficiency) + log(Sentence_reading_time) + Directional verb * sense * Congruency + (1 + Directional_verb + sense | participant) + (1 | stimuli).

^5^Model equation: log(RT) ~ log(Proficiency) + log(Sentence_reading_time) + Directional verb * Congruency + sense * Congruency + (1 + Directional_verb + sense | participant) + (1 | stimuli).

^6^Model formula: log(Sentence_reading_time) ~ log(Proficiency) + Directional_verb * Congruency + Sense * Congruency + (1 + Directional_verb + Sense | participant) + (1 | stimuli).

^7^Model equation: log(RT) ~ log(Age_of_acquisition) + log(Length_of_immersion) + log(Hours_of_English_communication) + log(IELTS) + log(IELTS_reading) + log(Sentence_reading_time) + Preposition * Congruency + Sense * Congruency + (1 + Preposition + Sense | participant) + (1 | stimuli).

^8^Model equation: log(RT) ~ log(Age_of_acquisition)*Congruency + log(Length_of_immersion)*Congruency + log(Hours_of_English_communication)*Congruency + log(IELTS)*Congruency + log(IELTS_reading)*Congruency + log(Sentence_reading_time)*Congruency + (1 | participant) + (1 | stimuli).

^9^Model formula: log(Sentence reading time) ~ log(Age_of_acquisition) + log(Length_of_immersion) + log(Hours_of_English_communication) + log(IELTS) + log(IELTS_reading) + Preposition * Congruency + Sense * Congruency + (1 + Preposition + Sense | participant) + (1| stimuli).
